# Catheter mechanoprophylaxis against Candida species

**DOI:** 10.1099/mic.0.001653

**Published:** 2026-01-14

**Authors:** Abhinay V. Adlooru, Walid K. Bibi, Paula A. Hernandez, Alexander M. Tatara

**Affiliations:** 1 Division of Infectious Diseases and Global Medicine, The University of Texas Southwestern Medical Center, 5323 Harry Hines Blvd, Dallas, Texas 75390, USA; 2 Department of Biomedical Engineering, The University of Texas Southwestern Medical Center, 6000 Harry Hines Blvd, Dallas, Texas 75235, USA; 3 Department of Orthopaedic Surgery, The University of Texas Southwestern Medical Center, 5323 Harry Hines Blvd, Dallas, Texas 75390, USA

**Keywords:** biofilm, biomaterial, *Candida*, catheter, catheter-associated urinary tract infection (CAUTI), central line-associated bloodstream infection (CLABSI)

## Abstract

*Candida* species infection of vascular and urinary catheters is a growing clinical concern. By understanding how biomaterial physicochemical surface properties affect fungal behaviour, catheters could be designed to mechanically discourage infection as a form of ‘mechanoprophylaxis’. In this study, silicone surfaces were synthesized with ‘stiff’ or ‘soft’ mechanical properties and the subsequent adherence, proliferation and biofilm production of *Candida albicans*, *Candida parapsilosis* and *Nakaseomyces glabratus* isolates on these surfaces were analysed. *Candida* significantly bound more, proliferated more and produced more biofilm on softer silicone surfaces. Importantly, the observed differences in fungal adhesion and biofilm formation between catheter surface types persisted when surfaces were pre-coated with host serum proteins. This study demonstrated that catheter synthesis parameters can affect physical properties and subsequent susceptibility to fungal colonization. These data lay important groundwork in exploiting mechanical design to decrease the ability of *Candida* to colonize devices and thus prevent medical device infections.

## Introduction

Biomedical devices have become ubiquitous in the practice of medicine. For example, 60–70% of hospitalized patients will have peripheral venous catheters, and 12–26 % will have a urinary catheter at any given time [1,2]. As foreign bodies, these devices are risk factors for infections, including central line-associated bloodstream infection (CLABSI) and catheter-associated urinary tract infection (CAUTI). In recent years, *Candida* species have become the most common organisms associated with CLABSI and CAUTI [3,4]. Invasive candidal infections have high rates of mortality, and multidrug-resistant isolates are a major threat to human health [5,7].

The interactions between fungi and catheter biomaterials are an important gap in our current understanding of pathogenesis and a potential opportunity for therapeutic intervention [8]. We have previously demonstrated that fungi such as *Mucorales* are responsive to mechanical signals in the environment and that these mechanical signals can drive virulence and increase mortality in *in vivo* models of disease [9]. If *Candida* mechanobiology was better understood, catheters could be designed with specific physicochemical properties that impair *Candida*’s ability to infect devices, as a form of ‘mechanoprophylaxis’.

Catheters are fabricated from materials including latex and silicones with properties that may affect adherence and colonization. For example, polydimethylsiloxane (PDMS), a silicone used in medical materials [10], can be prepared with different stiffnesses by controlling material crosslinking during synthesis. Multiple investigators have found that stiffer formulations of PDMS result in decreased attachment of *Escherichia coli*, *Pseudomonas aeruginosa* and *Staphylococcus aureus* [11,13]. Altering PDMS crosslinking also affects key pathogenic attributes such as biofilm deposition and subsequent bacterial susceptibility to antibiotics [13].

Despite the predilection of *Candida* species for catheter infection, little is known about the effect of silicone synthesis parameters on fungal behaviour. In a study of oral pathogens that included *Streptococcus mutans*, *Streptococcus oralis* and an odontogenic strain of *Candida albicans*, there was less adherence of all three species to PDMS with higher stiffness [14]. Interestingly, when surfaces were coated with saliva beforehand, the differences in *C. albicans* attachment were lost.

To further clarify the role of catheter synthesis parameters on physicochemical properties and subsequent *Candida* behaviour, we sought to measure *Candida* species attachment and biofilm production on soft and stiff PDMS surfaces with and without the coating of serum proteins. We used four well-defined strains. *C. albicans* SC5314 is a reference isolate originally derived from a patient with invasive candidiasis and produces a robust biofilm [15,16]. *C. albicans* CHN1 is a clinical isolate in a different evolutionary clade than SC5314 [17,18]. *Candida parapsilosis* Cp478 and *Nakaseomyces glabratus* ATCC2001 were chosen as other yeast species that cause catheter infections [19,21]. Unlike *C. albicans*, *C. parapsilosis* and *N. glabratus* do not produce true hyphae [19]. *N. glabratus* is considered particularly virulent and is often intrinsically resistant to azoles [21]. After characterizing the ‘stiff’ and ‘soft’ silicone catheter biomaterials, we studied candidal adherence, proliferation and biofilm production and measured the effect of surface coating with host serum proteins.

## Methods

### Reagents

PDMS (Sylgard 184 silicone elastomer kit, Electron Microscopy Sciences, Hatfield, PA), Sabouraud dextrose broth (SDB) (Difco, Franklin Lakes, NJ), Sabouraud dextrose agar (SDA) (Difco, Franklin Lakes, NJ), FBS (Thermo Fisher Scientific, Waltham, MA), crystal violet (Thermo Fisher Scientific, Waltham, MA), RPMI 1640 Medium (Thermo Fisher Scientific, Waltham, MA) and glacial acetic acid (Thermo Fisher Scientific, Waltham, MA). All reagents were used as received except for glacial acetic acid (diluted with diH_2_O to create 33% acetic acid), crystal violet (diluted with diH_2_O to create a 0.1% solution) and FBS (diluted with RPMI 1640 to create 10% FBS).

### Silicone fabrication

**Fig. 1.**
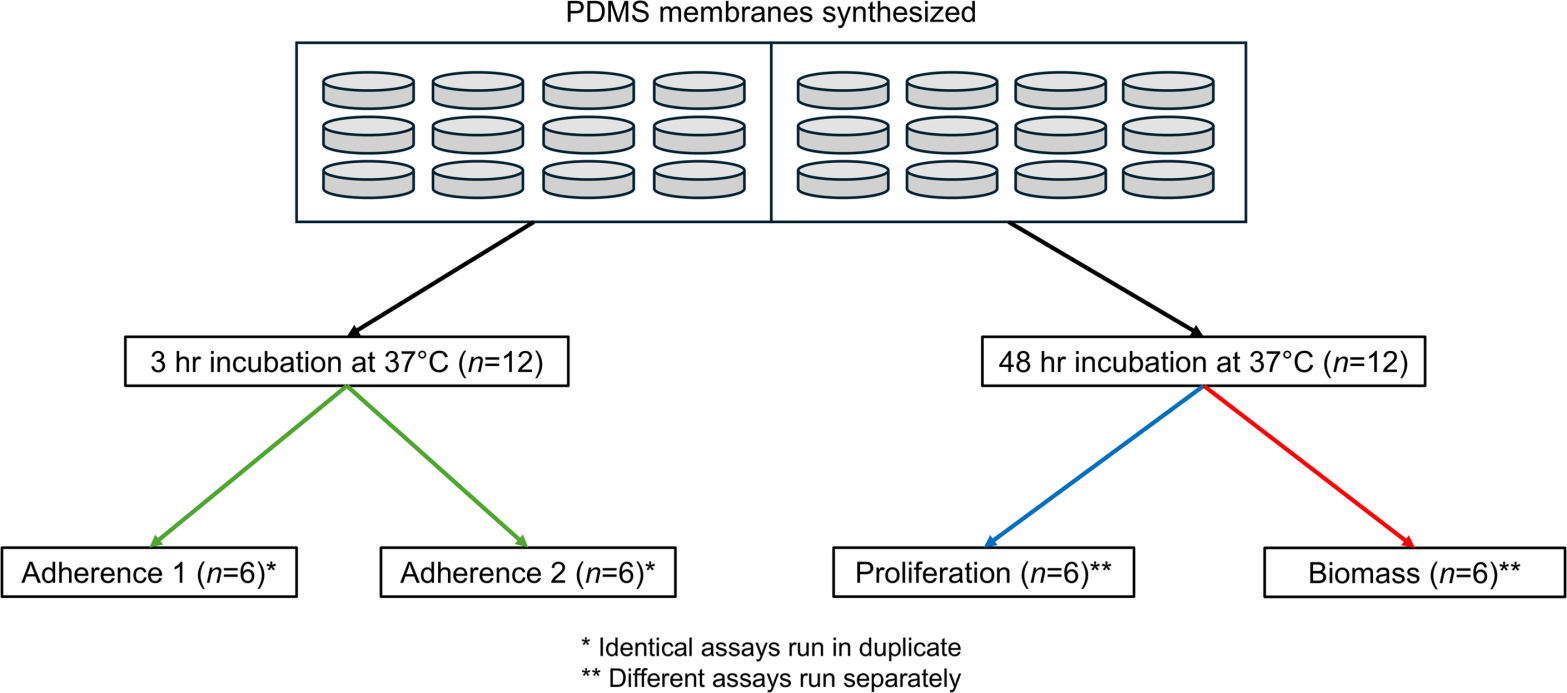
Flow chart of *in vitro* assays.

PDMS membranes of differing stiffness were synthesized based on established protocols [13,22]. PDMS base and curing agent were mixed at room temperature with base-to-curing agent ratios of 10:1 (‘stiff’) or 40:1 (‘soft’). Uncured PDMS solutions were added to glass slides with a 1-mm polytetrafluoroethylene spacer to create sheets of 1 mm in height or added to polytetrafluoroethylene moulds with blocks of dimensions 72 mm in length × 9 mm in width × 2.7 mm in height. Solutions were de-gassed for 45 min and cured at 60 °C for 3 h. For fungal assays, discs (4 mm in diameter × 1 mm in height) were produced with a biopsy punch from the resulting PDMS sheets. Prior to use in assays (Fig. 1), PDMS discs were sterilized via submersion in 70 % ethanol for 20 min, washed 2× in sterile diH_2_O and dried overnight in a sterile biosafety cabinet.

### Mechanical characterization

For mechanical characterization, blocks of dimensions 24 mm in length × 9 mm in width × 2.7 mm in height were cut and underwent tensile testing until failure with a crosshead velocity of 500 mm min^−1^ per the standard ASTM D412 [23] using a TA.HD*Plus Connect* Texture Analyzer (Texture Technologies, South Hamilton, MA) with Exponent Connect software to plot stress vs. strain for Young’s modulus measurement (*n*=16 per group).

### Wettability assessment

Surface wettability was assessed via contact angle measurements [24]. A 3 µl droplet of diH2O was placed onto the surface of sterilized discs (one droplet per disc with one measurement taken per droplet; *n*=5 per group). Images were taken with the lens 5 cm away from each droplet. The contact angle (the angle where the liquid droplet meets the disc surface) was manually measured with ImageJ (v1.54g) using the ‘Angle tool’ available as part of the base ImageJ functions.

### Scanning electron microscopy

Discs were sputter-coated with 2 nm of gold/palladium target with a Leica EM ACE600 sputter coater (Wetzlar, Germany) and then imaged with a Zeiss Sigma VP field emission scanning electron microscope (Oberkochen, Germany) at an accelerating voltage of 3 kV. For surfaces exhibiting buckling, the distance between buckled edges (*n*=20) was measured using ImageJ (v1.54g).

### Atomic force microscopy

Calculations of surface roughness were done using topographic images obtained with an MFP-3D Bio Atomic Force Microscope (Oxford Instruments Asylum Research, Santa Barbara, CA). Samples were scanned in air with a silicon nitride cantilever (Olympus AC240TS-R3 probe, Oxford Instruments Asylum Research) with a nominal spring constant *k*~2 N/m. The cantilever was calibrated using GetReal, which is based on the Sader method [25]. The same cantilever was used for all samples, scanning an area of 20×20 µm at a rate of 0.3 Hz with a 90° angle and a set point of 1.5 V. Roughness measurements (Ra, average roughness, and Rq, root mean square roughness) were captured as an average of ~six measurements using the Igor Pro v6.38 software (Oxford Instruments Asylum Research). Three discs from each group underwent testing.

### *In vitro* fungal assays

Single colonies of yeast (*C. albicans* SC531, *C. albicans* CHN1, *C. parapsilosis* CP478 or *N. glabratus* ATCC2001) were isolated on SDA plates and added to 5 ml of SDB for overnight incubation at 37 °C and 200 r.p.m. An 8×10^5^ c.f.u. ml^−1^ dilution was created by measuring optic density per McFarland standard, and concentration was confirmed the following day by plating and c.f.u. counting.

For adherence assays, 100 µl of 8×10^5^ c.f.u. ml^−1^ *Candida* was added to each PDMS disc in individual wells on a 96-well plate and incubated at 37 °C for 3 h (*n*=6 repeated on two different days for a total of 12 per group [26,27]). To assess proliferation and biofilm production, the assay was repeated at a timepoint of 48 h (*n*=6 repeated on 2 different days for a total of 12 per group; split into *n*=6 each for c.f.u. counting or biofilm biomass staining subgroups). To remove adherent yeast, discs were washed 2× in sterile diH_2_O, suspended in 1 ml of SDB with 0.3% Tween 80, sonicated (Cole-Parmer UC-200, Vernon Hills, IL) for 10 min at 4 °C and vortexed for 5 min at 4 °C following 3 h or 48 h of incubation [28]. The resulting media then underwent standard serial dilution and plating on SDA plates for c.f.u. counting. To understand the impact of adsorbed host proteins on fungal attachment, 10% solutions of FBS were made in RPMI 1640. Sterile discs were incubated with or without 100 µl of 10% FBS at 37 °C for 1 h [29]. Discs were washed with SDB ×1 prior to inoculation to remove unadsorbed proteins. Adherence and biofilm assays were repeated in the presence of adsorbed protein.

For further analysis of biofilm formation following 48 h of incubation, discs were washed 3× in sterile diH_2_O to remove non-adherent fungi, stained with 0.1% crystal violet solution in diH_2_O and washed 6× with diH_2_O to remove excess stain not absorbed by the biofilm biomass. Biofilms were allowed to dry overnight while protected from light. Prior to biofilm extraction, discs were imaged with a Zeiss Axioplan 2 microscope (Oberkochen, Germany) at 20× magnification to obtain representative brightfield images. Bound crystal violet was then extracted with 33% acetic acid [30]. The optical density of the extracted stain was measured at 595 nm (BMG LabTech CLARIOstar Plus, Cary, NC) using BMG LabTech MARS software.

### Statistics

All statistics were performed in R (v4.1.1). Data were tested for normality via Shapiro–Wilk’s test (*α*=0.05), and all data sets were found to be normal. Comparisons of two groups were made via the Welch two-sample t-test. Comparisons of more than two groups were made via ANOVA with Tukey’s honestly significant difference (HSD) post hoc. For all statistics, *α* was 0.05.

## Results

### Physicochemical characterization

**Fig. 2.**
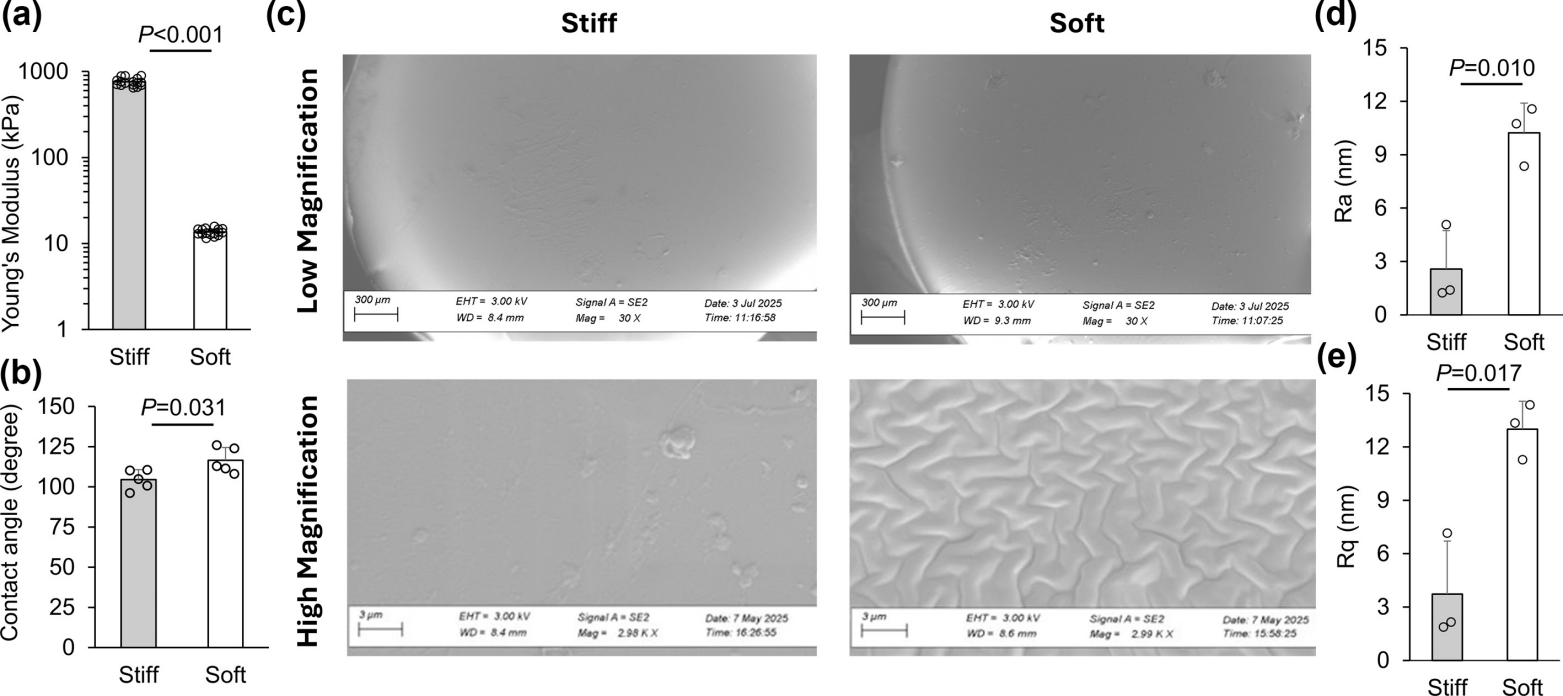
Physicochemical characterization of stiff and soft silicone surfaces. (**a**) Young’s modulus (*n*=16/group). (**b**) Contact angle measurements (*n*=5/group). (**c**) Representative scanning electron micrographs under low magnification (top, scale bar=300 µm) and high magnification (bottom, scale bar=3 µm). (**d**) Atomic force microscopy average roughness (Ra) (*n*=3/group). (**e**) Atomic force microscopy root mean square roughness (Rq) (*n*=3/group). Groups were compared via a Welch two-sample t-test. Bars represent mean values, and error bars represent standard deviation.

Soft and stiff silicones underwent mechanical tensile testing (*n*=16 per group) per parameters specified by an industry standard for the evaluation of thermoplastic elastomers (American Society for Testing Materials or ASTM Standard D412) [23]. The Young’s modulus (*E*) is a measurement of stiffness; it reflects how much force is required to deform a material. Stiffer materials require more force to deform and therefore have a higher *E*. For these PDMS devices, *E* ranged from 646.9 to 893.8 kPa for stiff PDMS and 11.5 to 15.1 kPa for soft PDMS (Fig. 2a). Stiff PDMS had significantly greater *E* than that of soft PDMS (753.5 vs. 13.6 kPa, *P*<0.001). Stiff and soft catheter surfaces underwent contact angle characterization (*n*=5 per group) (Fig. 2b), a method used to measure material hydrophobicity/wettability by a liquid [24]. Soft PDMS had a greater contact angle (104.5° vs. 116.5^o^°, *P*=0.031), indicating greater hydrophobicity and lower free surface energy [31,32].

Scanning electron micrographs showed minimal differences between surface types at low magnification (Fig. 2c, top). However, under high magnification, stiff silicone surfaces had a smooth topography, whereas soft silicone surfaces exhibited a ‘buckled’ texture (Fig. 2c, bottom). The mean distance between buckled edges was 1.46 µm with a standard deviation of 0.29 µm (*n*=20). Atomic force microscopy was performed to further characterize the roughness of the two surfaces. The average roughness and root mean square roughness were both significantly higher for the soft silicone surface (Fig. 2d–e).

### *Candida* adhesion and proliferation

**Fig. 3.**
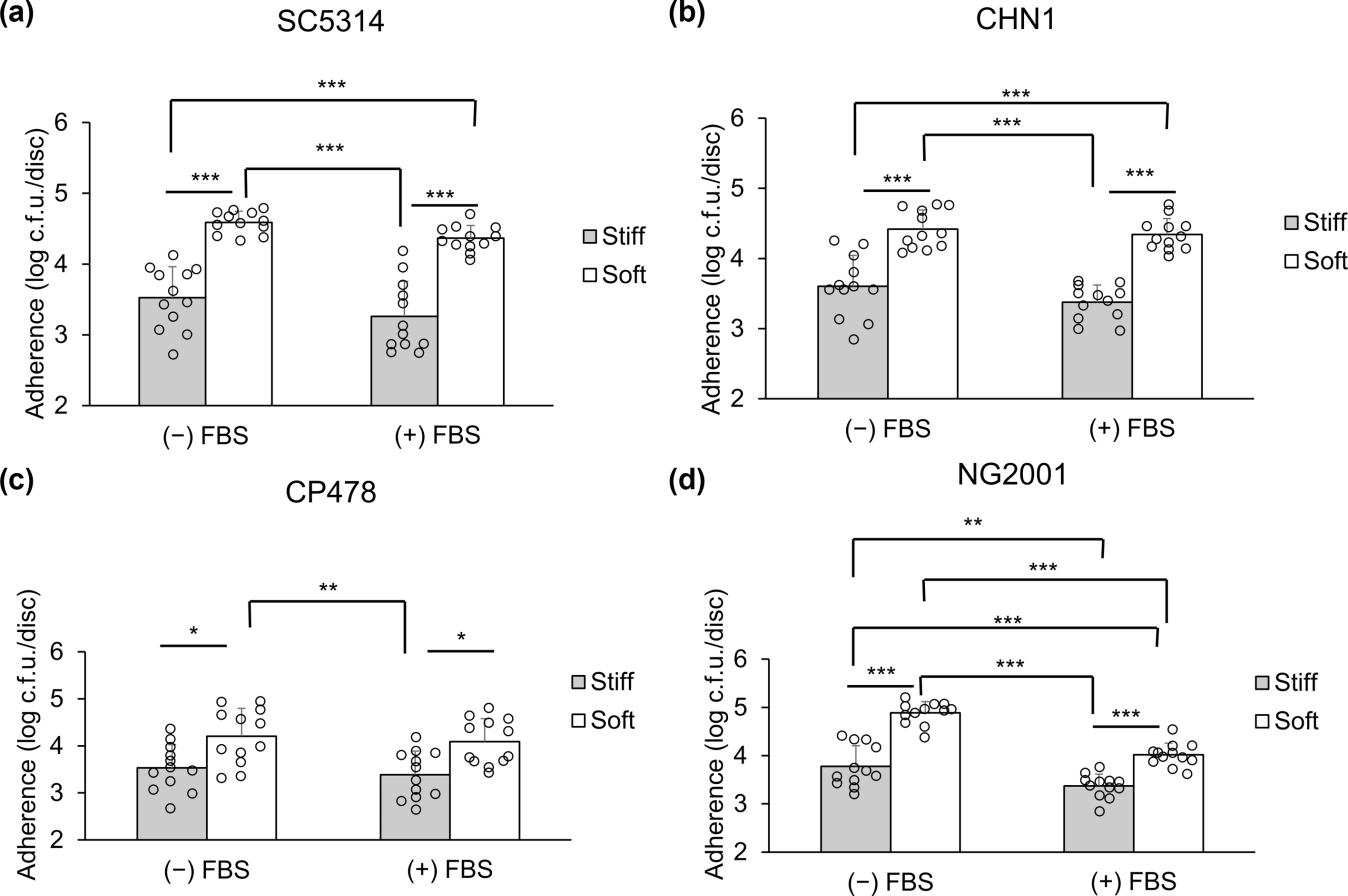
Adherence to stiff and soft silicone surfaces after 3 h of incubation across four different yeast strains *[C. albicans* SC5314 (**a**), *C. albicans* CHN1 (**b**), *C. parapsilosis* Cp478 (**c**), and *N. glabratus* ATCC2001 (**d**)] with (+) FBS precoating or without (−) FBS precoating (*n*=12 per group). Groups were compared via two-way ANOVA with Tukey’s HSD test. *P<0.05, **P<0.01 and ***P<0.001. All *P* values are listed in Table S1. Bars represent mean values, and error bars represent standard deviation.

Following 3 h of incubation at 37 °C, all four yeast strains had significantly less adherence to stiff silicone compared to soft silicone in the absence of any mammalian proteins (Fig. 3, Table S1, available in the online Supplementary Material). We also studied whether the presence of host serum proteins disrupted the ability of silicone physicochemical properties to affect fungal adherence and colonization [14]. Silicone surfaces were pre-coated with adsorbed fetal bovine serum [(+) FBS] [29]. For all species, there continued to be significantly greater adherence to pre-coated soft surfaces compared to pre-coated stiff surfaces. When directly comparing across a single type of surface with and without FBS, *C. albicans* strains and *C. parapsilosis* attachment were not affected by adsorbed protein. For *N. glabratus*, pre-coating discs with FBS resulted in significantly less binding to both soft and stiff discs (Fig. 3d).

**Fig. 4.**
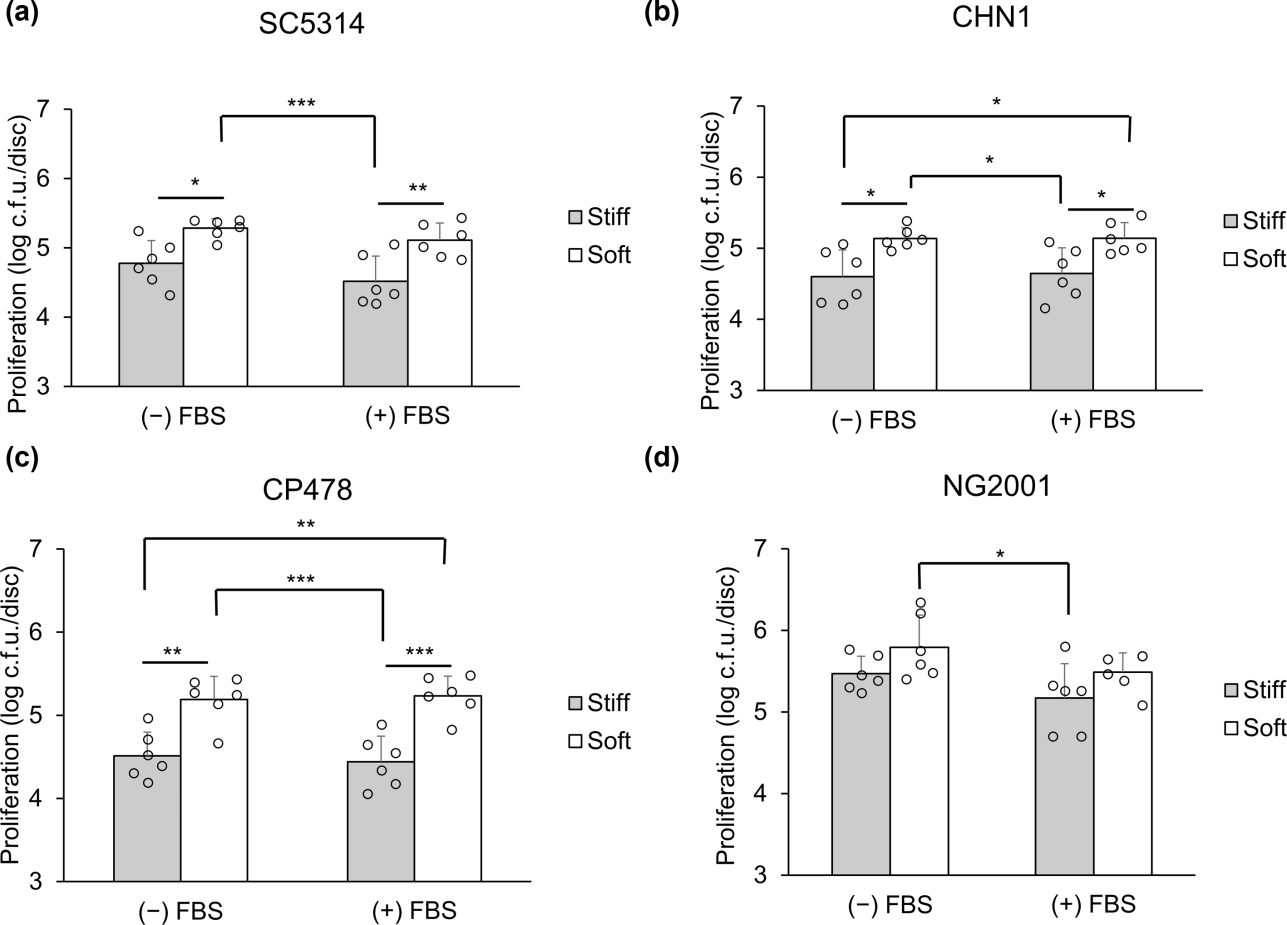
Number of adherent organisms on stiff and soft silicone surfaces after 48 h of incubation across four different yeast strains [*C. albicans* SC5314 (**a**), *C. albicans* CHN1 (**b**), *C. parapsilosis* Cp478 (**c**), and *N. glabratus* ATCC2001 (**d**)] with (+) FBS precoating or without (−) FBS precoating (*n*=6 per group). Groups were compared via two-way ANOVA with Tukey’s HSD test. *P<0.05, **P<0.01 and ***P<0.001. All *P* values are listed in Table S2. Bars represent mean values, and error bars represent standard deviation.

**Fig. 5.**
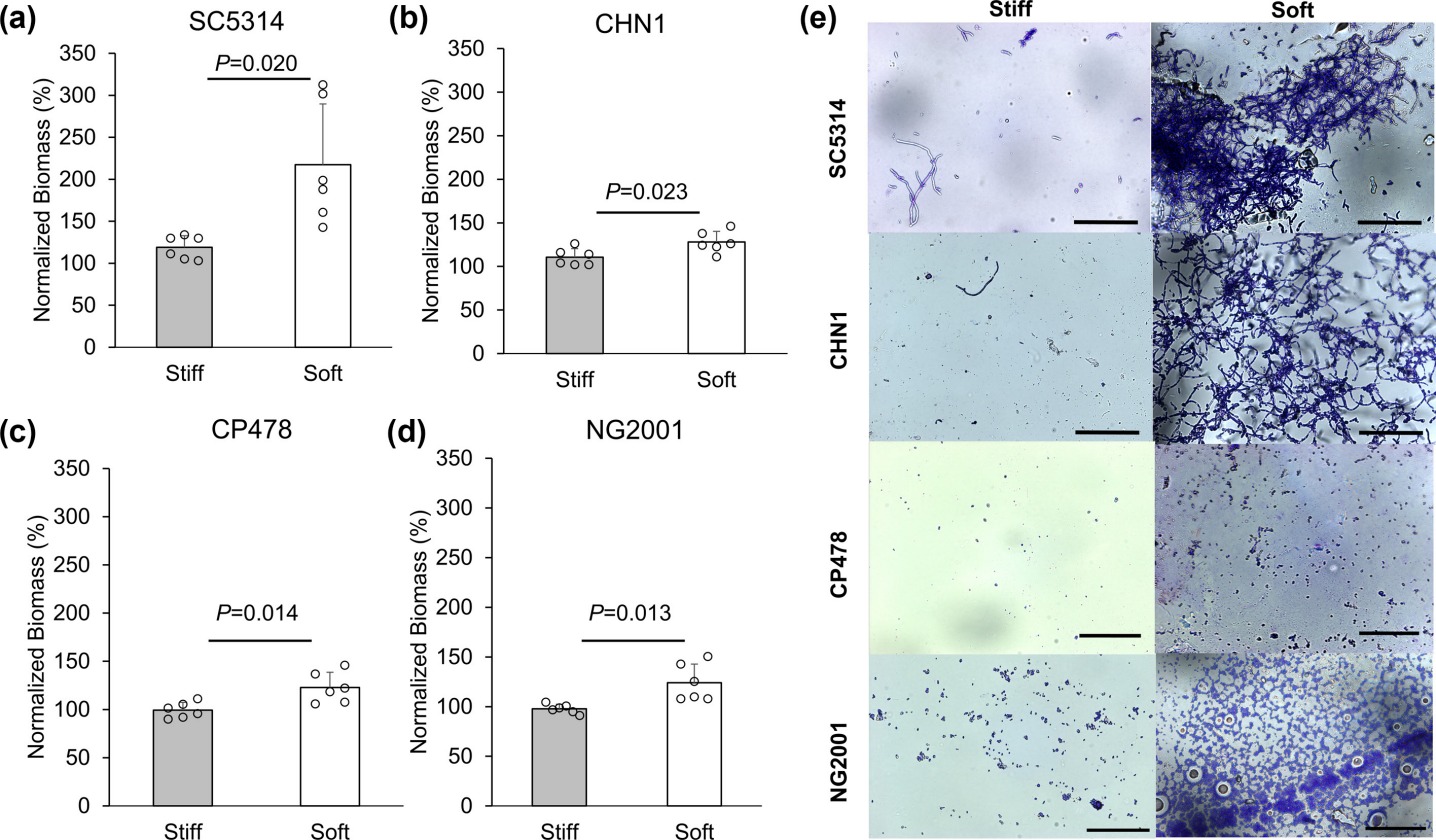
Biofilm biomass assays after 48 h of incubation on stiff and soft silicone surfaces. (**a–d**) Percent biomass normalized to stained sterile silicone discs for *C. albicans* SC4314 (**a**), *C. albicans* CHN1 (**b**), *C. parapsilosis* Cp478 (**c**) and *N. glabratus* ATCC2001 (**d**) (*n*=6/group). (**e**) Representative bright field micrographs of crystal violet-stained biofilm biomass (scale bar=500 µm). Surfaces were compared via Welch two-sample t-test. Bars represent mean values, and error bars represent standard deviation.

Following 48 h of incubation, the number of yeast colonies on all surfaces increased with and without FBS pre-coating (Fig. 4, Table S2). On stiff uncoated surfaces, *N. glabratus* had a significantly more rapid increase in c.f.u. between timepoints compared to the other species and had a more rapid increase on soft membranes compared to *C. albicans* CHN1 and *C. parapsilosis* CP478 (Table S3). At 48 h, there were significantly greater biofilm-embedded fungi recovered from soft silicone surfaces compared to stiff surfaces for *C. albicans* and *C. parapsilosis* (Fig. 4a–c). There was a trend towards greater c.f.u. on softer surfaces for N. *glabratus,* but this was not statistically significant for (−) FBS or (+) FBS groups (Fig. 4d). Staining with crystal violet demonstrated significantly more biofilm biomass produced on the soft silicone surfaces after 48 h of incubation (Figs 5 and S1).

## Discussion

In this study, we explored the effect of silicone synthesis parameters (base to crosslinker ratio) on surface physicochemical properties and subsequent biomaterial/pathogen interactions among four different strains of *Candida*. This expands the current literature, which has primarily focused on bacterial pathogens. We chose PDMS as our model medical silicone as it is well-explored in bacterial and mammalian mechanobiology [11,13,22].

Stiffness (Fig. 2a) was the most dramatic property modified by altering crosslinking density during synthesis (more than 55-fold greater *E*). Consistent with prior work in bacteria, *Candida* adhered and proliferated more readily to the soft silicone (Figs 3 and 4). There was also significantly greater biofilm biomass production on soft silicone surfaces (Fig. 5). One hypothesis is that adhesion to softer surfaces causes less cell envelope stress compared to the surface deformation required to adhere to stiff surfaces [33].

While the two different synthesis parameters significantly affected adhesion (Fig. 3) and biofilm biomass production (Fig. 5) across all four species of *Candida*, there were no differences in *N. glabratus* proliferation at 48 h (Fig. 4d). Of note, *N. glabratus* had ~10× (one log) greater c.f.u. compared to the other three species by 48 h, which may speak to its virulent nature [21]. It had a significantly greater rate of increase between the study’s two time points compared to the other isolates (Table S3). This baseline rapid growth rate may confound a relatively small effect size driven by differences in surface types at this late time point.

Silicone wettability, measured by contact angle°, increases with increasing base to crosslinker ratio during PDMS synthesis and correlates with greater hydrophobicity and decreased surface free energy [32]. Hydrophobic–hydrophobic interactions may promote more adhesion between *Candida* and abiotic hydrophobic surfaces. Regulation of cell surface hydrophobicity is a virulence mechanism of *C. albicans* and is modulated through increased expression of hydrophobic cell surface proteins such as Csh1p [34]. In this study, both stiff and soft surfaces would be categorized as hydrophobic, given a water contact angle greater than 100° with the softer group as more hydrophobic [35]. While statistically different, the 12° difference (Fig. 2b) is likely biologically marginal. In general, contact angle measurements are poor at predicting biological responses to materials [36]. Furthermore, material hydrophobicity was not uncoupled from stiffness and surface topography in this study.

Despite synthesizing both formulations of PDMS in the same moulds, the ‘soft’ formulation had a ‘buckled’ topography (Fig. 2c) with greater surface roughness (Fig. 2d–e). Others have appreciated a rough buckled topography with soft silicones and attribute these features to intrinsic buckling due to surface instability during synthesis [32]. Classically, rougher surfaces have been believed to promote more candidal adherence due to penetration into surface cracks [37]. However, recent research suggests factors such as surface free energy (related to wettability) have a more profound effect on *Candida* adherence [31]. In fact, surface topography can inhibit *Candida*; features 2 µm or smaller have been shown to inhibit *C. albicans* SC5314 adherence and biofilm production [38]. This is speculated to be due to fewer available binding sites (disrupted by the topographical feature) and/or because adhesion to surfaces at these ratios smaller than the individual yeast/hyphae incurs a binding energy penalty. In the present study, the buckles in the soft silicone were ~1.5 µm wide, smaller than *C. albicans* yeast (~5–7 µm long), *C. albicans* hyphae (~2.5–3.5 µm in diameter), *C. parapsilosis* yeast (3.2–7.3 µm long) and *N. glabratus* yeast (~2.7–3.5 µm long) [39,41] so were less likely to be major drivers of fungal behaviour.

When medical devices are implanted in the body, they rapidly become coated with host serum proteins (the Vroman effect [42]). As pathogens can subsequently bind to these protein complexes as well as the surface itself, it was important to determine if differences in fungal interactions with silicone physicochemical properties persist in the presence of serum proteins. If there are no longer differences in fungal behaviour when host proteins have coated a material surface, then interactions at the fungal/biomaterial interface may not be consequential *in vivo*. A prior study focusing on oral pathogens found that pre-coating PDMS with saliva negated all previously appreciated differences in *C. albicans* adherence to soft vs. stiff silicone surfaces [14]. In the present study, we found that differences in adherence and proliferation on soft and stiff silicone surfaces were persistent even in the presence of serum proteins. As *Candida* is an omnipresent colonizer of the oral cavity but only pathologically in the bloodstream, it is possible that the salivary proteome contains factors that differentially affect *Candida* behaviour [43].

From a translational perspective, these findings may contribute to the design of catheters with ‘mechanoprophylaxis’ to better resist yeast colonization, although results will need to be validated in an *in vivo* system. Consistent with our findings, although not specific to fungal infection, a meta-analysis of urinary catheters with hydrophilic surface coatings resulted in fewer urinary tract infections compared to conventional hydrophobic catheters [44]. In our study, the stiff group has a Young’s modulus similar to current catheters [45]. In theory, catheters could be designed with even stiffer inner lumens. Citing patient comfort, some ureteral stents have been developed using softer biomaterials [46] – these data raise concerns regarding infection risk.

There are several key limitations to our study. This is a preliminary mechanistic study that examined silicone catheters fabricated using two different crosslinking densities, which modified catheter physical properties such as elasticity, as captured in the Young’s modulus. One limitation of the study is not being able to isolate Young’s modulus from other physical properties, such as roughness/topography and hydrophobicity (significant although numerically small differences), which can confound results. However, this does capture real-world limitations in the synthesis parameters of medical-grade silicones. There are also a variety of other physical properties that could be exploited for mechanoprophylaxis, such as viscoelasticity and free surface energy, which could be the focus of future work given these results. Further, shear stress induced by flow in catheters is another mechanical signal that is also known to stimulate *Candida* [47]. Another limitation is that our model was also monomicrobial. For indwelling urinary catheters, most biofilms are polymicrobial and feature both bacterial and fungal species, which may have further complex interactions regulating virulence [48,49]. Lastly, these experiments were performed entirely *in vitro*. This removes variability inherent to living systems and allows for a more facile assay. However, even with pre-coating surfaces with mammalian serum, the complexity of host biological factors cannot be recapitulated in an *in vitro* system, including the interactions between yeast and different circulating immune cells. The impact of silicone catheter physicochemical properties with these additional factors (host immunity, polymicrobial colonization and flow) on fungal infection will be the focus of future work, leveraging established murine models of catheter infection [50,51] with our silicone synthesis methods. Mechanoprophylaxis is only one possible tool to manage fungal catheter infections. Another area under active investigation is the use of enzymatic anti-biofilm agents [52].

## Conclusions

The physicochemical properties of stiff vs. soft silicone catheter surfaces affect the adhesion and biofilm production of *Candida* species. The effect of these properties is persistent in the presence of host serum proteins. These findings may help drive decision-making in catheter design. Future work, including *in vivo* studies, will further elucidate whether the modification of device physicochemical properties can act as prophylaxis to mitigate fungal infection.

## Supplementary material

10.1099/mic.0.001653 Uncited Supplementary Material 1.

## Abbreviations

CAUTI: catheter-associated urinary tract infection
CLABSI: central line-associated bloodstream infection
HSD: honestly significant difference
PDMS: polydimethylsiloxane
SDA: Sabouraud dextrose agar
SDB: Sabouraud dextrose broth
