# Supplementary material for: Catheter mechanoprophylaxis against Candida species
Source: Microbiology (Reading). 2026 Jan 14;172(1):001653. doi: 10.1099/mic.0.001653 (PMC13293290; doi:10.1099/mic.0.001653)
Supplement: Uncited Supplementary Material 1. [file mic-172-01653-s001.pdf]

Supplemental Data for Catheter Mechanoprophylaxis Against *Candida* Species

Supplemental Table 1. Three-hour CFU comparisons between groups via two-way ANOVA with Tukey's HSD Test as a function of silicone surface type and pre-coating with fetal bovine serum. SC5314 = *Candida albicans* SC5314; CHN1 = *Candida albicans* CHN1; CP378 = *Candida parapsilosis* Cp378; NG2001 = *Nakaseomyces glabratus* ATCC2001; FBS = Fetal Bovine Serum.

Comparison	SC5314 (p value)	CHN1 (p value)	CP478 (p value)	NG2001 (p value)
Stiff (-) FBS) – Soft (-) FBS	<0.001	<0.001	0.014	<0.001
Stiff (+) FBS – Soft (+) FBS	<0.001	<0.001	0.010	<0.001
Stiff (-) FBS – Stiff (+) FBS	0.271	0.277	0.903	0.009
Soft (-) FBS – Soft (+) FBS	0.417	0.934	0.948	<0.001
Stiff (-) FBS – Soft (+) FBS	<0.001	<0.001	0.057	<0.001
Soft (-) FBS – Stiff (+) FBS	<0.001	<0.001	0.002	<0.001

Supplemental Table 2. Forty-eight-hour CFU comparisons between groups via two-way ANOVA with Tukey's HSD Test as a function of silicone surface type and pre-coating with fetal bovine serum. SC5314 = *Candida albicans* SC5314; CHN1 = *Candida albicans* CHN1; CP378 = *Candida parapsilosis* Cp378; NG2001 = *Nakaseomyces glabratus* ATCC2001; FBS = Fetal Bovine Serum.

Comparison	SC5314 (p value)	CHN1 (p value)	CP478 (p value)	NG2001 (p value)
Stiff (-) FBS) – Soft (-) FBS	0.026	0.022	0.002	0.348
Stiff (+) FBS – Soft (+) FBS	0.008	0.038	<0.001	0.366
Stiff (-) FBS – Stiff (+) FBS	0.407	0.993	0.969	0.419
Soft (-) FBS – Soft (+) FBS	0.709	1.000	0.993	0.400
Stiff (-) FBS – Soft (+) FBS	0.209	0.022	0.001	1.000
Soft (-) FBS – Stiff (+) FBS	<0.001	0.040	<0.001	0.019

Supplemental Table 3. Differences in colony-forming units between 3 and 48 hours of culture. SC5314 = *Candida albicans* SC5314; CHN1 = *Candida albicans* CHN1; CP378 = *Candida parapsilosis* Cp378; NG2001 = *Nakaseomyces glabratus* ATCC2001; FBS = Fetal Bovine Serum. Statistical comparisons made via two-way ANOVA with HSD Test.

(-) FBS	Stiff (CFU)	Soft (CFU)	(+) FBS	Stiff (CFU)	Soft (CFU)
SC5314	4.74 +/- 0.35	5.18 +/- 0.18	SC5314	4.48 +/- 0.38	5.01 +/- 0.31
CHN1	4.53 +/- 0.43	5.04 +/-0.18	CHN1	4.61 +/- 0.39	5.05 +/-0.26
CP378	4.45 +/- 0.32	5.13 +/- 0.34	CP378	4.39 +/- 0.34	5.20 +/- 0.26
NG2001	5.46 +/- 0.22	5.71 +/- 0.45	NG2001	5.16 +/- 0.43	5.47 +/- 0.25
Comparisons	Stiff (p value)	Soft (p value)	Comparisons	Stiff (p value)	Soft (p value)
SC5314-CHN1	0.948	0.994	SC5314-CHN1	0.997	1.000
SC5314-CP378	0.774	1.000	SC5314-CP378	1.000	0.974
SC4314-NG2001	0.010	0.116	SC4314-NG2001	0.022	0.265
CHN1-CP378	1.000	1.000	CHN1-CP378	0.938	0.995
CHN1-NG2001	<0.001	0.019	CHN1-NG2001	0.110	0.396
CP378-NG2001	<0.001	0.060	CP378-NG2001	0.006	0.838

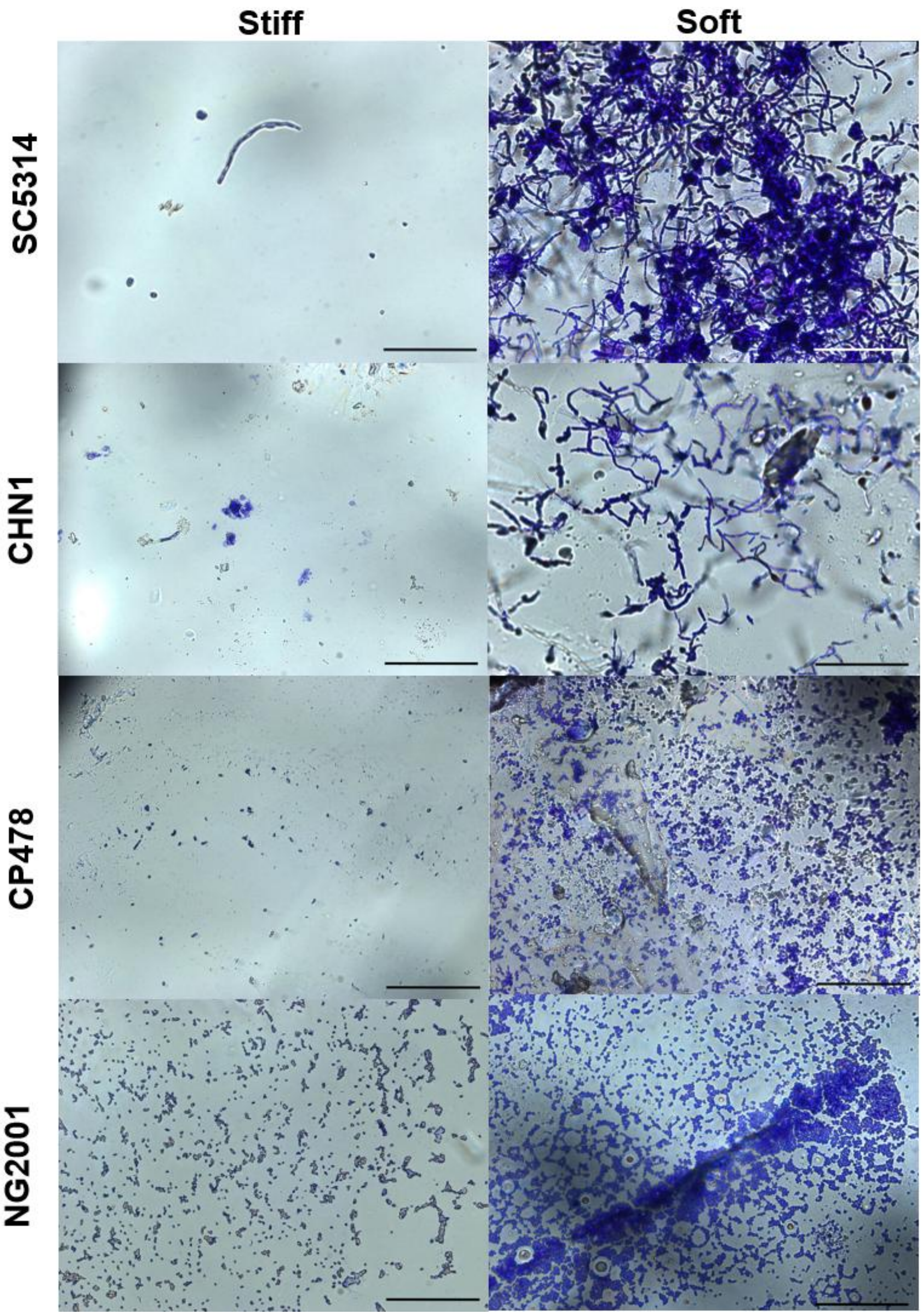

Supplemental Figure 1. Additional representative bright field micrographs of crystal-violet stained biofilm biomass (scale bar = 500 µm).
